# Phytochemical Profile, Antimicrobial, Cytotoxic, and Antioxidant Activities of Fresh and Air-Dried *Satureja nabateorum* Essential Oils

**DOI:** 10.3390/molecules27010125

**Published:** 2021-12-26

**Authors:** Nawaf Al-Maharik, Nidal Jaradat

**Affiliations:** 1 Department of Chemistry, Faculty of Sciences, An-Najah National University, Nablus P6028820, Palestine; 2 Department of Pharmacy, Faculty of Medicine and Health Sciences, An-Najah National University, Nablus P6028820, Palestine

**Keywords:** *Satureja nabateorum*, phytochemical profile, antibacterial, antifungal, antioxidant, anticancer

## Abstract

*Satureja nabateorum* (Danin and Hedge) Bräuchler is a perennial herb in the Lamiaceae family that was discovered and classified in 1998. This green herb is restricted to the mountains overlooking the Dead Sea, specifically in Jordan’s southwest, the Edom mountains, and the Tubas mountains in Palestine. Gas chromatography-mass spectrometry (GC-MS) analysis of essential oil (EO) of air-dried and fresh *S. nabateorum* resulted in the identification of 30 and 42 phytochemicals accounting for 99.56 and 98.64% of the EO, respectively. Thymol (46.07 ± 1.1 and 40.64 ± 1.21%) was the major compound, followed by its biosynthetic precursors *γ*-terpinene (21.15 ± 1.05% and 20.65 ± 1.12%), and *p*-cymene (15.02 ± 1.02% and 11.51 ± 0.97%), respectively. Microdilution assay was used to evaluate the antimicrobial property of EOs against *Staphylococcus aureus* (ATCC 25923), clinical isolate Methicillin-Resistant *Staphylococcus aureus* (MRSA), *Enterococcus faecium* (ATCC 700221) *Klebsiella pneumoniae* (ATCC 13883), *Proteus vulgaris* (ATCC 700221), *Escherichia coli* (ATCC 25922) and *Pseudomonas aeruginosa* (ATCC 27853) and *Candida albicans* (ATCC-90028). With a MIC of 0.135 μg/mL, the EOs has the most potent antibacterial action against *K. pneumonia*. Both EOs display good antifungal efficacy against *C. albicans*, with a MIC value of 0.75 μg/mL, which was better than that of Fluconazole’s (positive control, MIC = 1.56 μg/mL). The antioxidant capacity of EOs extracted from air-dried and fresh *S. nabateorum* was determined using the DPPH assay, with IC_50_ values of 4.78 ± 0.41 and 5.37 ± 0.40 μg/mL, respectively. The tested EOs showed significant cytotoxicity against Hela, HepG2, and COLO-205 cells, with IC_50_ values ranging from 82 ± 0.98 to 256 ± 1.95 μg/mL. The current work shows there is a possibility to use the *S. nabateorum* EOs for various applications.

## 1. Introduction

Medicinal aromatic plants have been utilized for their preservative and healing properties since ancient times. Some of these characteristics are attributed to essential oils (EOs), which are produced as plant secondary metabolites. The bulk of essential oils are composed of terpenes, but they also include non-terpene components such as aldehydes, alcohols, esters, phenols, ethers, and ketones [1]. Whose physical purpose is to protect plants against bacteria, fungus, viruses, insects, and even herbivores by reducing their appetite for these plants [2]. EOs have been utilized as antimicrobials, sedatives, food preservatives, food additives, and cosmetics [2,3]. More than 250 types of essential oils at a value of 1.2 billion USD are traded yearly on the global market. This is in line with a cultural shift away from synthetic medicines and toward natural treatments for a wide range of illnesses. As a consequence, numerous research groups are studying essential oils and their primary components for antioxidant, anthelmintic, antibacterial, antifungal, antiviral, anticancer, antiparasitic, and insecticidal activities [4,5,6].

Antimicrobial drug resistance caused by the massive use of antibiotics and the harmful side effects of synthetic antimicrobial drugs has prompted scientists to search for novel antimicrobial lead compounds. Numerous studies have documented the antibacterial and antifungal activities of EOs, their chemical components, and the synergistic impact of their components [6,7,8,9,10]. In this regard, the use of EOs derived from *Satureja* species may be an effective strategy for reducing bacterial resistance. Azaz et al. reported that EOs extracted from *Satureja hortensis*, *Satureja cuneifolia*, *Satureja thymbra*, *Satureja aintabensis*, and *Satureja macrantha* possess significant antibacterial and antifungal potential [11]. 

The Lamiaceae family, popularly known as mint or sage, is a well-known flowering plant family with 236 genera and 7630 species distributed worldwide [8]. The genus *Satureja* (*Thymbra*) is one of the large family’s genera, with approximately 200 species that grow naturally in the Middle East and Mediterranean European areas, as well as West Asia, North Africa, and South America [9]. *Satureja thymbra*, *Satureja thymbrifolia,* and *Satureja nabateorum*, of which the latter is only found in Palestine and Jordan, are the only three species of this genus known in Palestine. Thanks to the presence of physiologically active phytochemicals, *Satureja* EO affords the basis for a variety of biological and industrial applications with antimicrobial, antifungal, antioxidant, carminative, and digestive characteristics [12,13,14,15].

*Satureja nabateorum* Danin and Hedge is a 50–60 cm tall annual aromatic plant with white flowers. It is only found in smooth-faced sandstone crevices in Jordan’s southwest (Edom) and the mountains of Tubas, Palestine, near the Dead Sea. It is similar to *S. thymbrifolia*, a species found in the desert east of Jerusalem that reaches down to the Dead Sea and is part of the *S*. sect. *Zatarioideae* [16,17]. In Bedouin veterinary medicine, the *S. nabateorum* plant is used to treat livestock diseases. In continuation of our ongoing research program concerning the identification of phytochemical composition and the assessment of biological activities of various wild-growing herbs in Palestine, we have considered investigating the chemical composition of *S. nabateorum* EO and assessing its antimicrobial, free radical scavenging activity. To the best of the author’s knowledge, the phytochemical profile of *S. nabateorum* EO and its antimicrobial, antioxidant, and anticancer activity have never been studied before. Therefore, this research is aimed at (1) analyzing the chemical composition of EOs obtained from fresh and air-dried aerial parts of *S. nabateorum*, and (2) assessing their antioxidant, anticancer activity, and inhibitory activity on numerous microbial strains.

## 2. Results and Discussion

### 2.1. Chemical Composition of S. nabateorum Essential Oils

**Figure 1:**
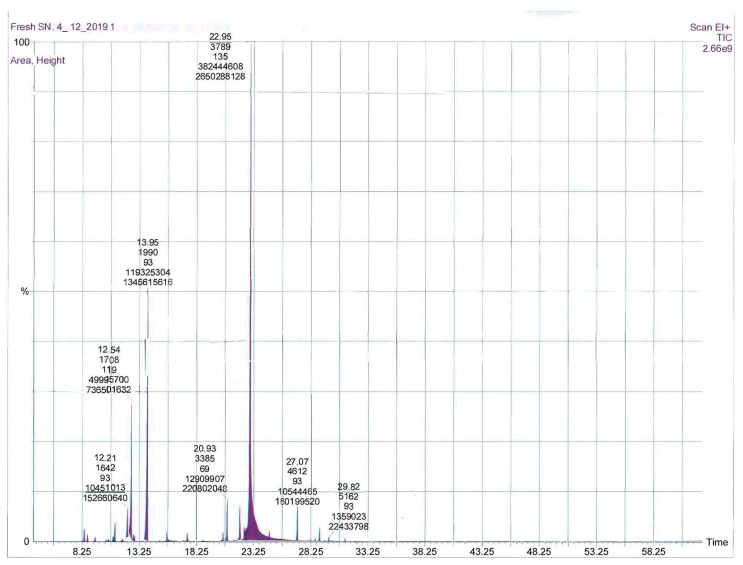
GC-MS chromatogram of the essential oils from fresh *S. nabateorum*.

**Figure 2:**
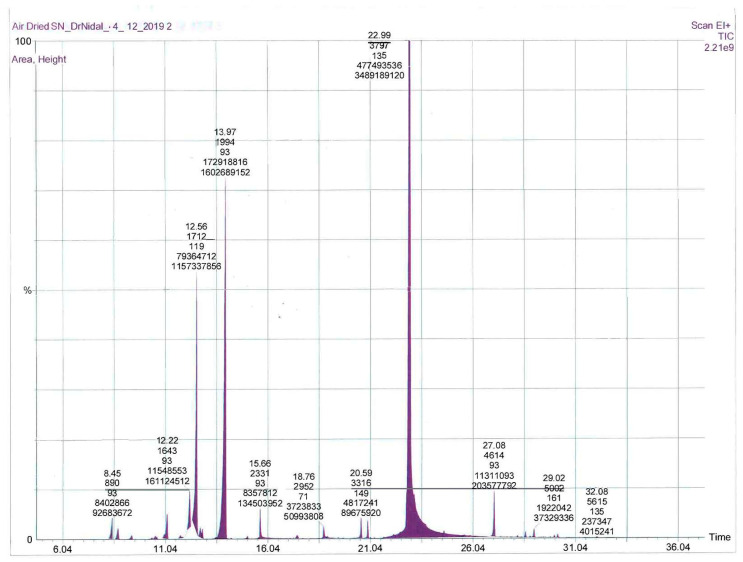
GC-MS chromatogram of the essential oils from air-dried *S. nabateorum*.

Microwave-assisted extraction of fresh and air-dried *S. nabateorum* aerial parts afforded light yellow oils. Table 1 contains the names, retention index, and percentages of identified compounds of both essential oils as obtained by GC-MS, as well as their analysis retention indices (RI). The GC chromatograms of EOs extracted from fresh and air-dried *S. nabateorum* are presented in Figure 1 and Figure 2. A variability in yields of the EOs obtained from fresh (1.80 ± 0.09%) and air-dried (1.55 ± 0.07%) *S. nabateorum* samples was observed, with minor chemical composition variation. However, the yield percentage was calculated using the following equation: Yield% = mass of EO/mass of plant × 100.

GC-MS allowed us to identify a total of 42 compounds in the investigated EOs of *S. nabateorum*, accounting for around 99% as relative to peak area, with thymol (46.07 ± 1.1 and 40.64 ± 1.21%), *γ*-terpinene (21.15 ± 1.05% and 20.65 ± 1.12%), and *p*-cymene (15.02 ± 1.02% and 11.51 ± 0.97%) being the main components of the EOs, respectively (Table 1 and Figure 1). Both EOs of *S. nabateorum* contained a high concentration of phenolic monoterpenes and monoterpenes (around 95%) and a very low concentration of sesquiterpenes. Significant differences were observed when the main components of *S. nabateorum* were compared to those of previously examined *Satureja* species. In contrast to other *Satureja* plants, carvacrol, a constitutional isomer of thymol, was not identified in the EO of *S. nabateorum*. Carvacrol was found to be the most prevalent component in most *Satureja* species’ EOs (27.24–88.71%), followed by *γ*-terpinene (3.18–23.20%) and *p*-cymene (2.77–13.34%) [17].

### 2.2. Antimicrobial Activity of EOs from Fresh and Air-Dried S. nabateorum 

In this investigation, EOs extracted from fresh and air-dried *S. nabateorum* display similar antibacterial activity against all tested microbial strains. As shown in Table 2, *S. nabateorum* EOs display greater antibacterial activity against *K. pneumonia*, *S. aureus*, *E. faecium*, and *E. coli* (MIC = 0.135–2.25 μg/mL) than ampicillin (MIC = 1.00–3.12 μg/mL), an antibiotic used to prevent and cure a variety of bacterial infections. The investigated EOs, on the other hand, exhibit antibacterial activity against *P. aeruginosa*, and MRSA clinical isolate with MIC values ranging from 6.25–12.50 μg/mL, which was comparable to that of ciprofloxacin (MIC = 3.12–12.50 μg/mL). EOs obtained from an air-dried sample exhibit greater antibacterial activity against MRSA (MIC = 6.25 μg/mL) than ciprofloxacin (MIC = 12.5 μg/mL). 

Giweli et al. reported that *S. thymbra* EO exhibits antibacterial activity against *B. cereus, M. flavus, S. aureus, L. monocytogenes, E. coli, P. aeruginosa, P. mirabilis,* and *S. typhimurium* with MIC values ranging from 0.001–0.1 mg/mL [18]. Furthermore, Markovic et al. [19] reported that *S. thymbra* EO displayed greater antibacterial activity than streptomycin (positive control) against Gram-positive bacteria (*B. cereus*, *S. aureus*, *M. flavus*, and *L. monoytogenes*) and Gram-negative bacteria (*P. aeruginosa*, *E. faecalis*, *S*. *Typhimurium*, and *E. coli*) with MIC values ranging from 0.6–5.0 μg/mL, of which *B. cereus* was the most sensitive bacteria and *L. monoytogenes* was the most resistant bacteria strain. Chorianopoulos et al. reported that *S. thymbra* L., *S. spinose*, and *S. parnassica* ssp. Parnassica possess antibacterial properties that outperform *Origanum* and *Thymus* species EOs against five foodborne bacteria, namely *E. coli*, *S. enteritidis*, *S. aureus*, *L. monocytogenes*, and *B. cereus* [20].

Both tested EOs display substantial fungicidal activity against *C. albicans*, with MICs values of 0.75 μg/mL, which was better than that of the positive control, fluconazole (MIC = 1.56 μg/mL). Geweli et al. reported that *S. thymbra* EOs display very strong antifungal activities with MIC values ranging from 1.00–25.00 μg/mL against *P. funiculosum*, *P. ochrochloron, A. flavus*, *A. ochraceus, A. niger, A. fumigatus, T. viride*, and *C. albicans* [18], of which *P. ochrochloron* was the most sensitive fungus, with a MIC value of 1 μg/mL, whereas *C. albicans* (MIC = 25 μg/mL) was the most resistant species. EOs used in this investigation outperformed *S. thymbra* in terms of antimicrobial activity. This significant antibacterial and antifungal efficacy may be attributed to the high concentrations of thymol (46.07%), γ-terpinene (21.15%), and *p*-cymene (15.02%), as well as to their synergistic impact. Chorianopoulos et al. found that *S. thymbra* EO, which contains only 42.39% thymol and carvacrol, possess higher antimicrobial activity against bacteria and fungi as opposed to EOs containing 88.71% thymol and carvacrol, such as *Origanum vulgare* ssp. hirtum (Link) [17]. This suggests that the antimicrobial activity is not purely attributed to the high percentage of these phenols, but that other minor active constituents play a part as well.

### 2.3. Antioxidant Activity

**Figure 3:**
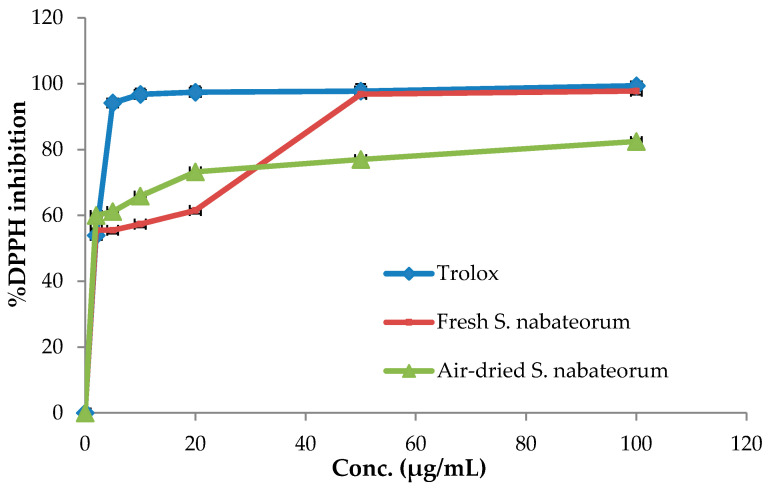
DPPH inhibitory activity by fresh and air-dried *S. nabateorum* EOs and Trolox.

The antioxidant property of fresh and air-dried *S. nabateorum* EOs was determined using the DPPH free radical scavenging assay, which is widely used to estimate the antioxidant potentials of plant EOs. Trolox, an artificial antioxidant, was used as a positive control. The findings showed that both EOs extracted from fresh and air-dried *S. nabateorum* have a dose-dependent free radical scavenging property (Figure 3).

The antioxidant outcomes revealed that air-dried *S. nabateorum* EOs display high antioxidant potential, as seen from the concentrations at which 50% of radicals have been quenched (IC_50_ = 4.78 ± 0.41 μg/mL), being slightly better than the antioxidant activity of EO extracted from fresh *S. nabateorum* aerial parts (IC_50_ = 5.37 ± 0.40 μg/mL) and weaker than that of Trolox (IC_50_ = 2.23 ± 1.57 μg/mL). The current study’s observations are in agreement with those of Elgndi et al. [21], who found that *S. montana* EO has significant antioxidant activity with an IC_50_ value of 5.05 ± 1.52 μg/mL. Aghbash et al. reported that *S. macrantha* EOs at various growth stages show remarkable oxidative activities ranging from 12.14 ± 0.63–21.85 ± 1.02 μg/mL, which are less active than *S. nabateorum* [22]. Geweli et al. documented that *S. thymbra* EO possesses antioxidant activity with an IC_50_ value of 96.7 μg/mL, which is much weaker than that of *S. nabateorum* EO [18]. Ghotbabadi et al. [23] assessed the antioxidant capacity of *S. sahendica* EOs at three phenological stages and found that EO obtained at the pre-flowering stage has the highest radical scavenging activity with an IC_50_ value of 7.85 ± 0.06 μg/mL, being slightly weaker than that of *S. nabateorum* EOs. 

The existence of phenolic monoterpenes thymol and carvacrol, as well as their precursors *γ*-terpinene and *p*-cymene, which behave synergistically as radical scavengers, is compatible with the radical scavenging activity of *Satureja* EOs [24,25]. EO extracted from air-dried *S. nabateorum*, in particular, exhibits higher potential antioxidant activity than the *Satureja montana* EO (5.05 ± 1.25 μg/mL) which is almost the same potentials as the current study fresh *S. nabateorum* EO (5.37 ± 0.4 μg/mL) [21].

### 2.4. Cytotoxic Characters

The cell viability of tested EOs was evaluated by the cells’ ability to metabolically convert MTT to a formazan dye after 24 h of exposure to EO at different doses ranging from 0.025 to 6.4 mg/mL. The cytotoxic activity (IC_50_ values) of both tested *S. nabateorum EOs* are shown in Table 3. Both essential oils display cytotoxic activity against MCF-7, COLO-205, HeLa, and HepG2 cancer cell lines in a dose-dependent manner, with IC_50_ values ranging from 82 ± 0.98 to 1090 ± 3.25 μg/mL (Table 3). EOs obtained from fresh *S. nabateorum* exhibit the highest cytotoxic effect against HeLa and HepG2 cancer cells, with IC_50_ values of 82 ± 0.98 and 90 ± 0.77 μg/mL, respectively, while EOs extracted from air-dried *S. nabateorum* exhibit the highest cytotoxic action against HeLa cancer cells, with IC_50_ values of 89 ± 0.74 and134.3 ± 1.2 μg/mL, respectively. Elgndi et al. [21] reported that *S. montana* EO, which is mostly comprised of carvacrol (57%) has cytotoxic action against HeLa cells with an IC_50_ value of 88.25 ± 10.85 μg/mL, which is consistent with the findings of the present investigation. According to Yousefzadi et al., *S. khuzistanica* EO significantly reduced cell viability of MCF7 cells in a dose-dependent manner, with an IC_50_ value of 125 μg/mL, which was better than *S. nabateorum* EOs. The cytotoxic effect is believed to be due to the high amount of carvacrol, which accounts for 93% of the total composition of *S. khuzistanica* EO. Yousefzadi found that *S. sahendica* EO exhibits significant cytotoxicity against MCF7, Vero, SW480, and JET 3 with IC_50_ values of 15.6, 15.6, 125, and 250 μg/mL^−1^, respectively [26].

## 3. Materials and Methods

### 3.1. Plant Material 

Aerial parts of *S. nabateorum* were gathered from the Jericho mountains in Palestine, which overlook the Dead Sea, before blooming in May 2019. The plant’s identity was verified by Dr. Nidal Jaradat and the voucher specimen herbarium marked Pharm-PCT-1633 was kept in the Herbal Products Laboratory at An-Najah National University-Nablus-Palestine. The plant was washed with distilled water, dried in the shade for two weeks at 25 ± 2 °C and 55 ± 5 RH humidity, milled with a mechanical grinder, and stored in paper bags.

### 3.2. Extraction of the Essential Oils 

The EOs were extracted from fresh and air-dried aerial parts of *S. nabateorum* using the Ultrasonic-Microwave apparatus, as previously described for other Lamiaceae species [27]. A 1L round-bottom flask containing a suspension of dried plant powder (50 g) in distilled water (0.5 L) was placed in the apparatus and attached to the Clevenger device. The microwave power was set at 800 W, and the EO was extracted for 20 min at 90 °C. The experiment was repeated three times under the same conditions. The combined extracts were dried with calcium chloride and stored in a sealed vial at 2–8 °C. 

### 3.3. Qualitative and Quantitative Analysis

For gas chromatographic experiments, a Perkin Elmer Elite-5-MS fused silica capillary column (30 m × 0.25 mm, film thickness 0.25 m) was used. The helium flow rate was set at 1.1 mL/min. The injector temperature was set at 250 °C, and the oven temperature set to 50 °C for 5 min before ramping up to 280 °C, at a rate of 4.0 °C/min. The total run time was 62.50 min, with a solvent delay of 0 to 4.0 min. The MS spectra in scan mode was between minute 4 to minute 62.5 and had a mass range of 50.00 to 300.00 *m*/*z*. The composition percentages were determined using the area data and rounded to the second decimal place. The components of *S. nabateorum* EO were detected by comparing their respective Kovats retention index (RI) values to those published in the literature, as well as by comparing the components’ mass spectra (M^+^, and fragmentation pattern) to those in the National Institute of Standards and Technology (NIST08) MS data Center, and by co-injection with authentic compounds such as thymol and carvacrol. n-Alkanes (C_7_–C_30_) mixture was employed for the determination retention indices (RI). 

### 3.4. Antimicrobial Screening

#### 3.4.1. Microbial Isolates

A broth microdilution method was utilized for evaluation of the antibacterial property of *S. nabateorum* EOs against seven bacterial strains which were obtained from the American Type Culture Collection (ATCC) and one diagnostically confirmed species. The Gram-negative strains were *Pseudomonas aeruginosa* (ATCC 9027), *Escherichia coli* (ATCC 25922), *Klebsiella pneumonia,* (ATCC 13883), and *Proteus vulgaris* (ATCC 8427). Gram-positive strains included *Enterococcus faecium* (ATCC 700221), *Staphylococcus aureus* (ATCC 25923), and Methicillin-Resistant *Staphylococcus aureus* (MRSA). The antifungal activity of the investigated EOs was evaluated against the growth of *C. albicans* (ATCC 90028).

#### 3.4.2. Antimicrobial Test

Using broth microdilution, the antibacterial efficacy of fresh and air-dried *S. nabateorum* EOs was determined [28]. The studied microorganisms were cultured separately on sterilized Muller-Hinton broth for 24 h at 37 °C using the streak plate process. A test tube containing 5 mL of nutrient broth was inoculated with bacterial strain from overnight colonies under aseptic conditions. The microorganism suspension was shacked in the test tubes. The turbidity of the bacterial suspension was modified to meet the turbidity standard 0.5 McFarland (1.5 × 10^8^ cfu/mL) and was subjected to three-fold dilution by mixing 1 mL of the suspension with 2 mL of the nutrient broth to achieve turbidity of 0.5 × 10^8^ cfu/mL. The Turbidity of the bacterial suspension was measured at 600 nm. 

The EOs were dissolved separately in 10% DMSO (Riedel-De-Han, Germany) to a concentration of 200 μg/mL. Each of the two solutions was serially micro-diluted (2-fold) in sterile Mueller Hinton broth. Next, 100 μL of Mueller Hinton broth was placed into each well of the 96-well microtiter plate (Greiner bio-one, North America), followed by the addition of 100 μL of the EO dilution to wells of the first column, making the total volume in each well of the first column 200 μL. Thereafter, starting from the first column, serial dilutions up to column #11 were performed by transferring 100 μL of dispensed suspension from the previous to the next well, and 100 μL of the suspension was removed from the 11th column. Wells in column 12 were kept free from EOs and considered as a positive control. Subsequently, 1 μL of the bacterial suspension was added to each well except wells #11, which was considered a negative control for microbial growth. Finally, the plate was incubated for 24 h at 37 °C (Incubator-Nuve, Turkey). For all bacteria tested in the current work, ciprofloxacin, ampicillin, and fluconazole were used as positive controls in the current study. The EOs were tested in triplicate.

### 3.5. Free Radical Scavenging Activity

The antioxidant activity of the two EOs was assessed based on their ability to scavenge the free radical diphenyl-1-picrylhydrazyl (DPPH) [1]. A stock solution (1 mg/mL) was prepared from *S. nabateorum* EOs by dissolving 100 mg of each EO in methanol (100 mL), of which serial dilutions to obtain different concentrations (1, 2, 5, 10, 20, 50, and 100 μg/mL) was made. Next, 1 mL of the diluted EO and 1 mL of methanol were mixed with 1 mL of DPPH methanol solution (0.002%). After 30 min incubation at room temperature in a dark place, the absorbance of the samples was recorded at 517 nm against the control using a UV-visible spectrophotometer. A blank solution was prepared by replacing the EO solution with neat methanol. The inhibition percentage was calculated according to the following equation.

$$I\;\left(\%\right)=\frac{\left[\mathrm{ABSblank}\;-\;\mathrm{ABStest}\right]}{\left[\;\mathrm{ABSblank}\right]}\times 100\%$$

The IC_50_ (μg/mL) values were calculated as the concentration, that scavenges 50% of DPPH radical. The same conditions were used for evaluating the inhibition percentage and IC_50_ of Trolox as a positive control. The antioxidant activity of the EOs was conducted in triplicate.

### 3.6. Cell Culture and Cytotoxicity Assay

The breast (MCF-7), liver (HepG2), cervix (HeLa), and colon (COLO 205) cancer cells were cultured separately in RPMI-1640 media, which was supplemented with 1% l-glutamine (Sigma, UK), 1% Penicillin/Streptomycin, and 10% fetal bovine serum. The cells were fully grown in a humidified environment with 5% CO_2_ at 37 °C and seeded at 2.6 × 10^4^ cells/well in a 96-well plate. After 24 h, cells were incubated with various concentrations of the tested EOs for 24 h. Cells viability test was assessed by CellTilter 96^®^Aqueous One Solution Cell Proliferation (MTS) Assay according to the manufacturer’s instructions (Promega Corporation, Madison, WI, USA). At the end of the treatment, 20 μL of MTS solution per 100 μL of media was added to each well and incubated for 2 h at 37 °C. Absorbance was measured at 490 nm. 

### 3.7. Statistical Analysis

All the conducted experiments were carried out in triplicate and the obtained results were expressed as mean ± SD and the results were considered significant when the *p*-value was less than 0.05.

## 4. Conclusions

The GC-MS analysis of the fresh and air-dried *S. nabateorum* EOs revealed that thymol was the major component, followed by γ-terpinene and p-cymene. Both oils exhibited potential antioxidant capacity and cytotoxic activity against HeLa and HepG2 cancer cells. In addition, the investigated oils showed remarkable antimicrobial effect, which may provide a potential alternative to chemical fungicides and bactericides, as well as antioxidants, and could find applications as natural preservatives and conservation agents in the food and/or cosmetic industries. Briefly, the investigated two EOs revealed a slight variation in the phytochemical compositions and biological activities. Further studies are required to isolate the biologically active compounds from these oils and to approve these effects in vivo and investigate their mechanism of action.

## Figures and Tables

**Table 1:** Chemical constituents of the essential oils from fresh and dry *Satureja nabateorum*.

	Component	RI	Dry, %	Fresh %
1	α-Thujene	918	1.24 ± 0.01	1.06 ± 0.12
2	α-Pinene	930	0.65 ± 0.002	0.59 ± 0.008
3	Camphene	952	0.23 ± 0.006	0.37 ± 0.005
4	Sabinene	972	0.08 ± 0.001	0.15 ± 0.001
5	β-Pinene	979	0.18 ± 0.001	0.20 ± 0.02
6	Isolimonene	984	-	0.41 ± 0.002
7	Myrcene	991	1.47 ± 0.01	0.69 ± 0.001
8	δ-2-Carene	1003	0.23 ± 0.002	0.25 ± 0.02
9	α-Terpinene	1019	2.81 ± 0.01	2.68 ± 0.98
10	p-Cymene	1023	15.02 ± 1.02	11.51 ± 0.97
11	Sylvesterine	1029	0.68 ± 0.009	0.60 ± 0.001
12	Ocimene	1033	0.60 ± 0.008	0.51 ± 0.006
13	*γ*-Terpinene	1061	21.15 ± 1.05	20.65 ± 1.12
14	Terpinolene	1087	-	0.09 ± 0.002
15	Linalool	1098	1.69 ± 0.05	0.78 ± 0.006
16	cis-Thujone	1103	-	0.17 ± 0.003
17	3-Terpinen-1-ol	1133	-	0.13 ± 0.001
18	(-)-Camphor	1144	-	0.69 ± 0.006
19	Not identified	1148	-	0.19 ± 0.003
20	Terpinen-4-ol	1173	0.69 ± 0.006	0.17 ± 0.002
21	Nerol	1228	-	0.15 ± 0.001
22	Thymol methylether	1235	1.21 ± 0.01	0.76 ± 0.005
23	Carvacrol methylether	1242	1.04 ± 0.01	3.44 ± 0.01
24	Linalool acetate	1256	-	0.15 ± 0.001
25	Geranial	1262	0.24 ± 0.002	2.91 ± 0.01
26	Neomenthyl acetate	1266	0.24 ± 001	-
27	Isobornyl acetate	1278	-	1.80 ± 0.01
28	Thymol	1292	46.07 ± 1.1	40.64 ± 1.21
29	α-Cubebene	1345	-	0.89 ± 0.002
30	α-Cupaene	1377	-	0.25 ± 0.004
31	β-Cubebene	1383	-	0.25 ± 0.002
32	β-Caryophyllene	1424	2.78 ± 0.006	2.85 ± 0.02
33	α-Caryophyllene	1457	0.14 ± 0.003	0.16 ± 0.001
34	Geranyl propanoate	1471	0.38 ± 0.001	0.30 ± 0.01
35	Muurolene	1476	0.11 ± 0.006	-
36	Germacrene D	1482	0.54 ± 0.003	1.14 ± 0.07
37	α-Muurolene	1497	0.07 ± 0.001	0.13 ± 0.06
38	Germacrene A	1508	0.07 ± 0.007	0.36 ± 0.21
39	*γ*-Cadinene	1514	0.16 ± 0.006	0.12 ± 0.03
40	*δ*-Cadinene	1528	0.22 ± 0.001	0.09 ± 0.001
41	Germacrene B	1552	0.04 ± 0.001	0.26 ± 0.004
42	Caryophellene oxide	1580	0.07 ± 0.001	0.10 ± 0.002
	Total		99.56	98.64
	**Grouped Compounds**			
	Monoterpene hydrocarbons		47.58	47.20
	Phenolic monoterpenes		48.32	44.84
	Sesquiterpene hydrocarbon		3.66	6.60

**Table 2:** Antibacterial and antifungal properties of the essential oils of *S. nabateorum* (MIC values in μg/mL).

	*S. aureus*	*E. faecium*	*E. coli*	*P. aeruginosa*	*K. pneumoniae*	*P. vulgaris*	MRSA	*C. albicans*
Source	ATCC 25923	ATCC 700221	ATCC 25922	ATCC 27853	ATCC 13883	ATCC 700221	Clinically diagnosed	ATCC 90028
Fluconazole	ND	ND	ND	ND	ND	ND	ND	1.56
Ampicillin	3.12 ± 0.06	1.56 ± 0.01	3.12 ± 0.01	ND	1 ± 0.01	18 ± 0.91	ND	ND
Ciprofloxacin	0.78 ± 0.01	0.78 ± 0.01	1.56 ± 0.08	3.12 ± 0.1	0.125 ± 0.02	15 ± 0.68	12.50 ± 0.58	ND
Fresh *S. nabateorum* EO	0.80 ± 0.01	1.25 ± 0.03	2.25 ± 0.01	6.25 ± 0.61	0.135 ± 0.04	22 ± 1.02	12.50 ± 0.61	0.75 ± 0.05
Air-dried *S. nabateorum* EO	0.85 ± 0.01	1.25 ± 0.05	2.25 ± 0.02	12.50 ± 1.31	0.135 ± 0.01	22 ± 1.13	6.25 ± 0.97	0.75 ± 0.05

MIC: minimum inhibitory concentration (μg/mL); ND: Not detected.

**Table 3:** Cytotoxic activity IC_50_ values of the EOs obtained from fresh and air-dried *S. nabateorum* compared with Doxorubicin.

	IC_50_ (μg/mL) Values against Different Cancer Cell Lines			
Cells	MCF-7	COLO-205	HeLa	HepG2
Air-dried *S. nabateorum*	1090 ± 3.25	134.3 ± 1.2	89 ± 0.74	ND
Fresh *S. nabateorum*	930 ± 1.25	256 ± 1.95	82 ± 0.98	90 ± 0.77
DOX	3 ± 0.89	5 ± 0.78	9.5 ± 1.0	25.43 ± 1.73

## Data Availability

All data is contained within the article.

## Sample Availability

Samples of the compounds are not available from the authors.
